# Cardiomyopathies in Women

**DOI:** 10.14797/mdcvj.1368

**Published:** 2024-03-14

**Authors:** Cindy M. Martin

**Affiliations:** 1 Houston Methodist DeBakey Heart & Vascular Center, Houston, Texas, US

**Keywords:** heart failure, sex-related differences, heart disease in women, genetic cardiomyopathy, cardiovascular disorders of pregnancy

## Abstract

Heart failure affects over 2.6 million people in the United States. While women have better overall survival rates, they also suffer from higher morbidity as shown by higher rates of hospitalization and worse quality of life. Several anatomical differences in women’s hearts affect both systolic and diastolic cardiac physiology. Despite these findings, women are significantly underrepresented in clinical trials, necessitating extrapolation of data from males. Because women have sex-specific etiologies of heart failure and unique manifestations in genetic-related cardiomyopathies, meaningful sex-related differences affect heart failure outcomes as well as access to and outcomes in advanced heart failure therapies in women. This review explores these gender-specific differences and potential solutions to balance care between women and men.

## Introduction

**Figure 1:**
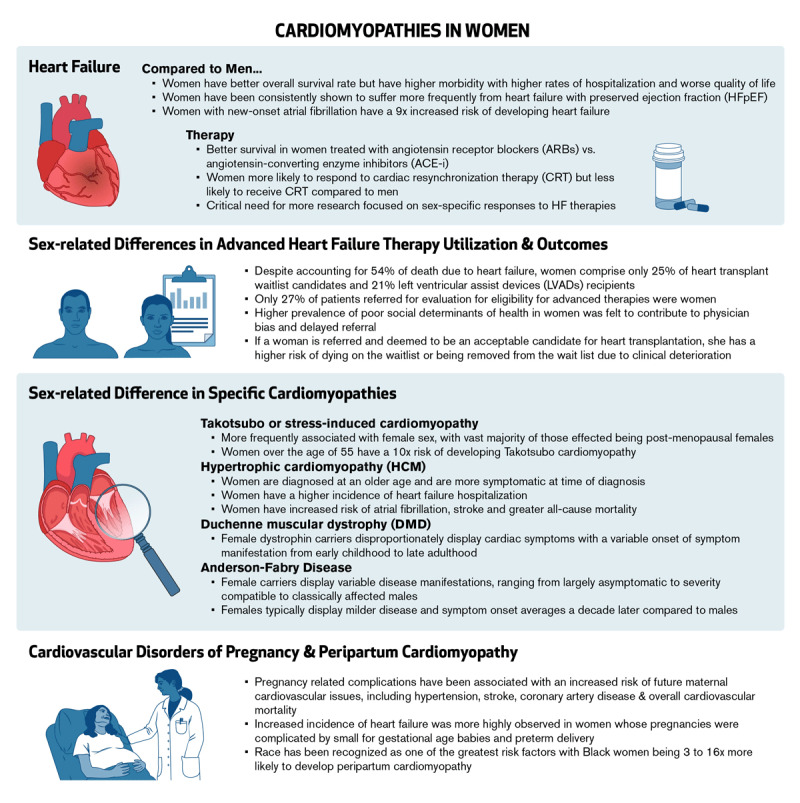
An overview of cardiopathies in women and sex-related differences in diagnoses, treatments, and outcomes.

Heart failure affects over 2.6 million individuals in the United States (US).^1^ Although overall survival rates are better in women, women suffer with higher morbidity as evidenced by higher rates of hospitalization and worse quality of life.^2,3,4,5^ Several anatomical differences have been discovered in women’s hearts, affecting both systolic and diastolic cardiac physiology. Women also have sex-specific etiologies of heart failure and unique manifestations in genetic-related cardiomyopathies (Figure 1). Emerging data highlights sex-related differences in both response to guideline-directed medical therapy for heart failure as well as in access to and outcomes for advanced heart failure therapies in women. Despite these findings, women are significantly underrepresented in clinical trials, necessitating extrapolation of data from males.

## Heart Failure

Heart failure (HF) has been defined as “a complex clinical syndrome with symptoms and signs that result from any structural or functional impairment of ventricular filling or ejection of blood.”^6^ Left ventricular (LV) cardiac function has been used to establish the classifications of HF. In 2022, based on new evidence, the American Heart Association (AHA)/American College of Cardiology (ACC) updated the classifications and definitions of HF as follows:

1. HF with preserved ejection fraction (HFpEF): left ventricular ejection fraction (LVEF) > 50%;
2. HF with reduced ejection fraction (HFrEF): LVEF < 40%; and
3. HF with mildly reduced ejection fraction (HFmrEF): LVEF between 41% and 49%.^6^

However, recent investigations show that women without evidence of heart disease have higher baseline LVEF than men, with the lower limit of normal being 55% in men but 61% in women.^7,8^ This difference may explain the varying outcomes in patients with LVEF ranging from 41% to 49% since an LVEF of up to 45% in women may behave more like HFrEF. Perhaps more importantly, the recognition that an LVEF of 55% may represent abnormal function in women is crucial, especially in monitoring female patients who have suffered myocardial infarction or who are undergoing potential cardiotoxic therapies such as chemotherapy.

Women are less likely to develop HFrEF compared with men, and the incidence of HF has declined more in women than in men.^9^ The etiology of HFrEF also demonstrates sex differences, with men more frequently having an ischemic etiology and atrial fibrillation, whereas women more frequently have a valvular disease etiology, hypertension, and diabetes.^10^ Interestingly, women with HFrEF exhibit less ventricular dilation, less fibrosis, and a lower risk of ventricular tachycardiac, which may partly account for the better survival seen in women with HFrEF compared to men.^9,11,12^

There also have been sex-related differences in response to medical therapy for HFrEF. Although multiple studies have demonstrated the overall benefit of angiotensin-converting enzyme inhibitors (ACEIs) in the treatment of HFrEF, two large meta-analyses showed no benefit of ACEI therapy in women with HFrEF.^13,14^ However, Val-HeFT (the Valsartan Heart Failure Trial) did demonstrate a reduced rate of hospitalization in women treated with valsartan.^15^ Additionally, a large Canadian observational study demonstrated better survival in women treated with angiotensin receptor blockers (ARBs) versus ACEIs, whereas no differences were noted in men.^16^ These findings raise questions about the benefit of the angiotensin receptor–neprilysin inhibitor (ARNI) compared with sacubitril–valsartan in women with HFrEF, especially given that an ACEI was used as the control instead of an ARB.

When analyzing nonpharmacologic HF treatments, women are shown to more likely respond to cardiac resynchronization therapy (CRT) with improved quality of life and ventricular remodeling and reduced HF hospitalizations and mortality.^17^ Yet women remain less likely than men to receive CRT.^18,19^ The overall benefit of implantable cardioverter-defibrillator (ICD) therapy in women remains somewhat questionable, as the landmark primary ICD trials enrolled few women. Two different meta-analyses of these trials failed to show a survival benefit for women. Although the risk of complications from ICDs remains low, women have consistently been found to have an increased risk compared with men.^20,21,22,23^ These findings, along with the fact that women with HFrEF have shown to have a lower risk of sudden cardiac death, emphasize the need for sex-specific trials in this area.

Studies show that women consistently suffer from HFpEF more frequently than men,^24^ although this is thought in part to be more strongly related to aging than simply female gender. Nevertheless, differences in cardiac physiology and aging in the female heart predispose to the development of HFpEF. Female hearts more frequently develop concentric remodeling.^25^ Women have increased higher systolic and diastolic elastance compared to men, and this difference further expands with increased age.^26^ Additionally, women display an increase in coronary microvascular dysfunction with aging compared to men, and this dysfunction plays a vital role in the development of HFpEF.^27,28^

Several cardiac-independent risk factors also increase the risk of HFpEF in women. Obesity has consistently been shown to be a significant risk factor for the development of HFpEF, and this effect is more pronounced in women.^29,30^ Atrial fibrillation is a common comorbidity associated with HF. In contrast to ventricular arrhythmias, more women than men experience atrial fibrillation.^31^ This increased incidence was traditionally attributed to the aging female population, but the recent Screening for Atrial Fibrillation Among Older Patients in Primary Care Clinics (VITAL-AF) clinical trial (ClinicalTrials.gov ID NCT03515057) revealed that when adjusted for height, women had a significantly higher risk of developing atrial fibrillation than men.^32^ Women with new-onset atrial fibrillation have a 9-fold increased risk of developing HF compared to men. Also, women with both HF and atrial fibrillation have significantly higher mortality than men with both conditions.^31^

Similar to HFrEF, men and women respond differently to medical therapies for HFpEF. A post-hoc analysis of the TOPCAT (Treatment of Preserved Cardiac Function Heart Failure with an Aldosterone Antagonist) trial demonstrated an improvement in all-cause mortality with spironolactone therapy in women but not men, although there was no difference noted in the primary outcome of composite cardiovascular mortality, aborted cardiac arrest, or HF hospitalization.^33^ PARAGON-HF (Efficacy and Safety of LCZ696 Compared to Valsartan, on Morbidity and Mortality in Heart Failure Patients With Preserved Ejection Fraction) also showed an improvement in the primary outcome in women but not in men. However, the benefit was mainly driven by women with LVEF between 45% and 60%, which―given the increased threshold of normal LVEF in females―may more strongly support the benefit of ARNI or simply ARB therapy in reduced LV function in women.^34^

## Sex-related Difference in Specific Cardiomyopathies

In addition to differences in development and treatment responses to both classifications of heart failure (HFrEF and HFpEF), sex-related differences also are seen in specific cardiomyopathies.

### Takotsubo Cardiomyopathy

Takotsubo (or stress-induced) cardiomyopathy is more frequently associated with female sex, with the vast majority of patients being post-menopausal females.^35^ Women over the age of 55 have a 10-fold risk of developing Takotsubo cardiomyopathy compared to men.^35^ Significant stressors are a common risk factor, although the types of stressors differ, with women more frequently presenting with psychological stressors while men develop cardiomyopathy in the setting of physical stressors.^35,36^ Men typically are younger but display more comorbidities compared to women.^37^ Men with Takotsubo cardiomyopathy also have an increased rate of HF and mortality compared with women.^36,38^

### Hypertrophic Cardiomyopathy

With hypertrophic cardiomyopathy (HCM), women are diagnosed at an older age than men, typically 6 to 9 years older, and they are more symptomatic at the time of diagnosis than men.^39,40,41^ Women with HCM also exhibit lower exercise capacity, even when controlling for age and gender, and have increased symptoms and limitations across all stages of the disease.^39,42^ Females with HCM have a higher incidence of HF hospitalization, increased risk of atrial fibrillation and stroke, and greater all-cause mortality, although no significant difference in sudden cardiac death is evident.^43^ Additionally, females with HCM show increased obstructive disease, smaller LV cavities, and increased diastolic dysfunction, yet fewer women use ICDs than men.^44,45^ A greater percentage of females with HCM have sarcomere gene variants,^41,46^ although penetrance of these gene mutations is threefold higher in men than women.^47,48^ Unfortunately, as commonly seen in more traditional forms of cardiomyopathy, females are consistently underrepresented in HCM studies.

### X-linked Cardiac Conditions

Cardiac manifestations of X-linked diseases were historically thought to primarily affect males. More recently, however, studies recognize that female carriers of many of these diseases also may exhibit significant disease manifestations, including cardiac involvement. Duchenne muscular dystrophy (DMD) is a fatal X-linked recessive condition caused primarily by out-of-frame mutations in the dystrophin gene. Becker muscular dystrophy (BMD) is a milder form of DMD typically caused by in-frame mutations of the dystrophin gene. In affected males, manifestations of DMD include profound progressive muscular weakness resulting in loss of ambulation and development of cardiomyopathy by adolescence. With improvements in respiratory therapies, cardiomyopathy has now surpassed respiratory failure as the leading cause of death in DMD patients.^49,50^

Additionally, heart failure is a significant cause of morbidity and mortality in patients with BMD.^51^ Female dystrophin carriers disproportionately display cardiac symptoms with a variable onset of symptom manifestation from early childhood to late adulthood.^52^ Cardiac involvement in female carriers may be subclinical under normal physiologic conditions but can manifest during times of cardiac stress, such as pregnancy.^52^ More recent studies reveal that with enhanced cardiac imaging screening, up to 40% of DMD carriers and more than 5% of BMD carriers were found to have LV dysfunction.^53^ In addition, up to 65% of DMD carriers and 20% of BMD carriers displayed myocardial fibrosis indicated by late-gadolinium enhancement on cardiac magnetic resonance imaging (MRI).^53^

Anderson-Fabry disease is a rare lysosomal storage disease caused by deficiency in alpha-galactosidase-A due to mutations in the GLA gene, leading to accumulation of glycosphingolipids in vital organs including the nervous system, gastrointestinal system, kidneys, and heart. Over half of patients with Anderson-Fabry disease have cardiac involvement, which is a significant cause of disease-related mortality and reduced life expectancy.^54^ Left ventricular hypertrophy is the most common cardiac manifestation, but arrythmias are also frequent.^55,56^ Despite being X-linked in inheritance, two-thirds of all patients with Anderson-Fabry disease are female.^54,57^ However, affected males display earlier onset with more severe disease manifestation. Female carriers display variable disease manifestations, ranging from largely asymptomatic to severity comparable to classically affected males. Females typically display milder disease, and symptom onset averages a decade later compared with men.^58^ Sex-specific therapies for X-linked cardiomyopathies are a needed area of study to help define when, how, and what specific type of therapy to begin in female carriers of X-linked cardiac conditions.

## Cardiovascular Disorders of Pregnancy and Peripartum Cardiomyopathy

Cardiac disorders of pregnancy are unique syndromes for women, and approximately 80% of females will have at least one pregnancy in their lifetime. Unfortunately, maternal morbidity and mortality is increasing in the US despite decreasing global rates.^59^ Up to 30% of singleton pregnancies are complicated by adverse pregnancy outcomes including preeclampsia, preterm birth, gestational diabetes mellitus, and small-for-gestational-age infants.^60^ In addition to increasing the risk for mother and infant morbidity and mortality during the pregnancy and early postpartum period, these pregnancy related complications have been associated with an increased risk of future maternal cardiovascular issues, including hypertension, stroke, coronary artery disease, and overall cardiovascular mortality.^60,61^

Regarding specific risk for heart failure, increased incidence of heart failure was more highly observed in women whose pregnancies were complicated by small-for-gestational-age babies and preterm delivery.^62^ Pregnancy is often referred to as a cardiovascular stress test, and thus the development of these cardiovascular disorders of pregnancy seems to unmask underlying risks of future cardiovascular disorders. More than 10 years ago, the AHA updated its guidelines for the prevention of cardiovascular disease in women to include cardiovascular disorders of pregnancy as a major risk for future cardiovascular disease, reinforcing the need to add pregnancy history to routine history and physical assessments for women.

Peripartum cardiomyopathy is a form of systolic heart failure that occurs toward the end of pregnancy or in the early postpartum period in the absence of other identifiable causes. A more specific definition has been proposed that defines the onset of heart failure as occurring in the last month of pregnancy or in the first 5 months of the postpartum period, with an LVEF of 45% or lower.^63^ However, some cases of peripartum cardiomyopathy are recognized to occur outside of these time ranges. While the cause of peripartum cardiomyopathy remains poorly understood, increasing evidence suggests a vascular etiology that is regulated by hormonal factors including prolactin, relaxin, activin A, and soluble Fms-like tyrosine kinase 1.^63^ A genetic predisposition for peripartum cardiomyopathy has been recognized for some time, and genetic variants seen recently in nonischemic cardiomyopathy also have been identified in up to 15% of patients with peripartum cardiomyopathy, with the majority of these being mutations in TTN, the gene encoding titin.^63,64^

Worldwide, peripartum cardiomyopathy complicates approximately 1 in 2,000 live births and is a leading cause of maternal death.^63^ Risk factors for development of peripartum cardiomyopathy include hypertension, preeclampsia, multiparity, and advanced maternal age.^65,66^ However, race is recognized as one of the greatest risk factors, with Black women being 3 to 16 times more likely to develop peripartum cardiomyopathy.^63,65,66,67,68^ They also take twice as long to recover cardiac function and are twice as likely to have persistently impaired cardiac function compared to White women.^63,66^ US mortality rates due to peripartum cardiomyopathy range from 7% to 20%, with the highest mortality rates seen in Black women.^69^

Currently, no specific therapies have proven beneficial for peripartum cardiomyopathy through randomized clinical trials, thus management strategies have been extrapolated from the guideline-directed medical therapy for HFrEF. If HF medical therapy is initiated prior to delivery, it must be tailored to avoid teratogenic effects to the fetus.^63,66,68^ Most standard HF medications can be utilized while breastfeeding; however, no safety information is currently available for newer agents such as sacubitril-valsartan or sodium-glucose cotransporter 2 inhibitors. Preliminary data supporting the use of bromocriptine to suppress prolactin release showed promise in the treatment of peripartum cardiomyopathy, but results from further clinical data were less clear.^63,66,70,71^ The ongoing Randomized Evaluation of Bromocriptine in Myocardial Recovery Therapy for Peripartum Cardiomyopathy (REBIRTH) trial, expected to be completed by 2026, should provide more guidance regarding the role of bromocriptine therapy. Given the hypercoagulable state of pregnancy, anticoagulation should be strongly considered if LVEF is less than 35% or atrial arrhythmias are also present in peripartum cardiomyopathy.^72,73^

Left ventricular systolic function at the time of diagnosis is the most reliable predictor of adverse events as well as cardiac recovery.^65^ Other factors associated with adverse outcomes include severe LV dilation, right ventricular systolic dysfunction, late gadolinium enhancement on cardiac MRI, prolonged QT intervals, and late diagnosis.^66,68^ Peripartum cardiomyopathy does have a significant overall rate of cardiac recovery, with 60% to 70% of patients demonstrating normalization of LV function.^74^ Recovery is typically seen within the initial 6 months but can occur as late as 2 years after diagnosis.^66,74^ Repeat pregnancies in women with a history of peripartum cardiomyopathy should be pursued cautiously. In cases where LV function has normalized, there is no significant increase in maternal death with subsequent pregnancies. However, there is a substantial risk of recurrent peripartum cardiomyopathy, thus successive improvement in cardiac function is not guaranteed.^63,66,68^ If LV function remains depressed in those with peripartum cardiomyopathy, the risk of maternal mortality with subsequent pregnancies can be as high as 20%.^75^ Discussions regarding further pregnancies in women with a history of peripartum cardiomyopathy are best done utilizing expert, multidisciplinary teams and shared decision making.

### Sex-related Differences in Advanced Heart Failure Therapy Utilization and Outcomes

Unfortunately, in all etiologies of HF, a percentage of individuals will continue to decline despite all currently available therapies. In these patients with advanced HF, therapeutic options are limited to heart transplantation and mechanical circulatory support devices. Despite accounting for 54% of deaths due to HF, women comprise only 25% of heart transplant waitlist candidates and 21% of LV assist device (LVAD) recipients.^76,77,78,79^ Some of this disparity can be explained by women more frequently suffering from HFpEF, which is less often amenable to LVAD or an indication for heart transplantation. However, this alone does not account for the massive sex-related difference in advanced HF therapy utilization. The road to advanced HF therapy utilization is a long one, and women must overcome hurdles at each step, starting with the first step of her physician recognizing advanced HF and referring to an advanced HF specialist.

In a recent multicenter retrospective analysis, only 27% of patients referred for evaluation of eligibility for advanced therapies were women.^80^ A 2022 manuscript by Ebong et al. highlighted several barriers to therapies for women, one of which was the higher prevalence of poor social determinants of health in women that was felt to contribute to physician bias and delayed referral.^81^ Increased personal caregiving responsibilities, actual or perceived inadequacy in social support, and mental health issues were recognized as other factors negatively impacting referral bias towards women.^81^ If a woman is referred and deemed an acceptable candidate for heart transplantation, she has a higher risk of dying on the wait-list or being removed from the wait-list due to clinical deterioration.^82,83^ Women are less likely to be bridged with an LVAD while awaiting transplant and also are less likely to receive temporary mechanical support devices (such as intra-aortic balloon pump, micro-axial LVADs, or extracorporeal membrane oxygenation) in the setting of cardiogenic shock, despite having a higher mortality rate in cardiogenic shock from HF compared to men.^83,84^ Women frequently have a higher degree of sensitization, which further lengthens the wait for transplantation and translates to a higher risk of antibody mediated rejection post-transplant.^82,83,85^ However, despite this, if a woman is able to make it to transplantation, long-term survival tends to be better in women than in men.^85^

As mentioned previously, the percentage of men receiving LVAD therapy for any indication is almost five times that of women.^79^ Although evidence is mixed, overall post-LVAD outcomes appear to be similar between the two genders.^81,86^ This again was supported by the sex-specific analysis of the MOMENTUM 3 study, of which 20.4% of participants were female with average age of 57.3 +/- 12.4 years, 44.2% Black race, and 23.9% ischemic etiology compared to 79.6% of participants who were male with an average age of 60.4 +/- 12.1 years, 23.6% Black race, and 49.6% ischemic in etiology.^87^ The analysis confirmed no difference between men and women in survival or the composite end point of survival free from disabling stroke or reoperation to replace or remove a malfunctioning pump at 2 years.^87^ Women, however, were shown to have a statistically significant increase in adverse events including stroke, gastrointestinal bleed, and major infection.^87^ This difference may be due in part to sex-derived hormonal and thrombotic/bleeding differences and/or sex-derived differences in pharmacodynamics of LVAD-associated anticoagulation and antiplatelet therapies.^88,89,90^ Interestingly, when the elderly subset (> 65 years of age) was studied, again there was no difference in 2-year survival or the composite end point between men and women but also no difference was seen in adverse events in this cohort.^87^ The complexity of these results again highlights the need for ongoing sex-specific research in both mechanical circulatory support therapies as well as transplantation outcomes.

## Conclusion

Although the annual rate of HF mortality is lower in females than in males, more women die from HF each year due to the increased overall incidence of HF in females.^91^ Women also have unique differences in presentation and progression in several types of cardiomyopathies, with peripartum cardiomyopathy and pregnancy-related cardiovascular conditions being conditions exclusive to females. Sex-related differences in response to HF therapies are beginning to be appreciated, but a critical need for further research in this area remains. Increased participation of underrepresented individuals, including women, has become a targeted focus in cardiovascular research. Efforts to augment the understanding of how gender, in addition to other factors such as race, ethnicity, and socioeconomic background, will allow future treatments to be individualized to provide enhanced benefit while limiting adverse events.

## Key Points

- Women with heart failure (HF) have better overall survival but have higher morbidity with higher rates of hospitalization and worse quality of life compared to men.
- Women have sex-specific etiologies of HF and display unique presentation and progression in several types of sex-shared cardiomyopathies.
- Sex-related differences in response to HF guideline-directed medical therapies are being increasing recognized.
- Despite accounting for the majority of deaths due to HF, women account for a quarter or less of those listed for heart transplantation or left ventricular assist device recipients.
- There is a critical need for more research focused on sex-specific therapies and outcomes in HF.
